# Correlation between Ankle Imaging Findings and Self-Reported Outcomes: A Longitudinal Assessment in Patients with Tibiofibular Diastasis

**DOI:** 10.3390/jcm12237239

**Published:** 2023-11-22

**Authors:** Samer Hosin, Dinu Vermesan, Bogdan Deleanu, Daniel Pop, Dan Crisan, Musab Al-Qatawneh, Mihai Mioc, Cosmin Faur, Ovidiu Rosca, Radu Prejbeanu

**Affiliations:** 1 Department of Orthopedics, “Victor Babes” University of Medicine and Pharmacy Timisoara, 300041 Timisoara, Romania; samerhosin@umft.ro (S.H.); dinu@vermesan.ro (D.V.); bogdandeleanu@yahoo.com (B.D.); daniellaurentiupop@yahoo.com (D.P.); crisan.dan@gmail.com (D.C.); msb898@gmail.com (M.A.-Q.); mihaillazarmioc@gmail.com (M.M.); faur.cosmin@umft.ro (C.F.); raduprejbeanu@gmail.com (R.P.); 2 Doctoral School, “Victor Babes” University of Medicine and Pharmacy Timisoara, 300041 Timisoara, Romania; 3 Department of Infectious Diseases, “Victor Babes” University of Medicine and Pharmacy Timisoara, Eftimie Murgu Square 2, 300041 Timisoara, Romania

**Keywords:** orthopedic procedures, tibiofibular ankle syndesmosis, ankle fractures, quality of life, joint range of motion

## Abstract

Background and Objectives: This longitudinal study investigated the correlation between imaging findings and self-reported questionnaire outcomes in patients with tibiofibular diastasis, exploring the effects of surgical screw removal versus conservative treatment. This study was conducted at “Victor Babes” University of Medicine and Pharmacy in Timisoara between 2018 and 2023. Materials and Methods: The study involved 85 patients in the screw removal group and 44 in the conservative group, assessed at 2 and 6 months post-surgery, answering the SF-36, HADS, and WHOQOL questionnaires. Results: Significant differences were observed at 2 months post-surgery, with the screw removal group showing lower shear wave velocities in ankle dorsiflexion (8.9 ± 1.4) and anterior talofibular ligament (2.8 ± 0.9), indicating better mobility compared to the conservative group (ankle dorsiflexion: 10.1 ± 1.8, ATFL: 3.2 ± 1.1). Radiographically, lower tibiofibular overlap (8.1 ± 2.1) in the screw removal group suggested improved joint fixation quality. These physical improvements were mirrored in the quality-of-life assessments, where the screw removal group reported higher physical health scores on the SF-36 survey at 2 months, a trend that continued at 6 months. At 2 months, ankle dorsiflexion demonstrated a strong negative correlation with the SF-36 Physical score (r = −0.417) and WHOQOL Physical domain (r = −0.394), and a positive correlation with HADS Anxiety (r = 0.312). Similarly, ATFL and CFL velocities negatively correlated with the SF-36 Physical score (ATFL: r = −0.251; CFL: r = −0.237). Radiographic tibiofibular overlap and clear space positively correlated with WHOQOL Physical domain (TOL: r = 0.291; TCS: r = 0.276), with TCS also negatively correlating with HADS Anxiety (r = −0.228). At 6 months, these correlations persisted, with notable negative correlations between ultrasound ankle dorsiflexion and both SF-36 Physical score and WHOQOL Physical domain. Conclusions: These findings underscore the advantages of screw removal in enhancing physical recovery and reducing anxiety in the short term, while indicating similar long-term mental health outcomes between treatment approaches.

## 1. Introduction

The ankle, a complex hinge joint, plays a pivotal role in human ambulation and overall lower extremity function. Its stability and biomechanical properties are sustained not only by the interplay of the involved bones but also by the multitude of ligaments and soft tissues surrounding it [1,2]. The distal tibiofibular syndesmosis is a focal point for many injuries, including tibiofibular diastasis, that can compromise ankle integrity, necessitating a thorough and accurate evaluation before clinical intervention [3,4].

Techniques such as X-ray, MRI, and CT scans have revolutionized the understanding and management of distal tibiofibular injuries, guiding both conservative approaches and surgical interventions [5,6,7,8,9,10,11]. Nevertheless, clinical evaluation, though vital, might not always correlate with patient experiences, functional outcomes, or their overall perception of well-being [12,13].

To bridge this gap, self-reported tools have gained attention in orthopedics, offering a nuanced understanding of the patient’s perspective [14,15]. These tools, encompassing structured questionnaires and scales, offer details about pain, mobility, daily activities, and quality of life, thereby providing a holistic view of patient recovery post-intervention [16]. Moreover, in the context of tibiofibular diastasis, while imaging delineates the structural attributes, self-reported metrics shed light on functional outcomes and the subjective experiences of the patients, such as quality of life metrics, physical disability scores, and pain levels that can affect the patient’s mental status and alter the recovery [17,18,19].

Although imaging findings and self-reported tools are individually beneficial, a correlation between the two might usher in a more comprehensive outlook on tibiofibular diastasis and its consequences. The synthesis of objective imaging data with the subjective experiences of patients can offer a profound understanding, potentially guiding better management strategies and enhancing patient satisfaction [20]. However, the existing literature largely studies these factors separately, with minimal focus on establishing a direct correlation [21,22].

Therefore, we hypothesize that there is a significant correlation between ultrasound and radiographic findings of the ankle and self-reported quality of life in patients with tibiofibular diastasis. The primary objective of our research is to explore the association between ultrasound and radiographic findings identified at 2 and 6 months after the orthopedic intervention, with the results provided from the patient-filled surveys regarding their quality of life, physical, and mental health.

## 2. Materials and Methods

### 2.1. Study Design and Settings

The current research was designed as a prospective longitudinal study and was executed between October 2018 and July 2023 at the University Clinic of Orthopedics affiliated with the “Victor Babes” University of Medicine and Pharmacy in Timisoara. This timeframe was chosen to ensure a sufficient number of cases for analysis and to observe longitudinal outcomes. The hospital’s electronic database was filtered to obtain relevant demographic information, clinical data, and imaging findings. All patient information was treated with the utmost confidentiality, adhering to prevailing privacy regulations, and only accessible to the certified medical staff involved in the study. The department strictly follows ethics regulations, governed by national and international laws on medical research involving human participants, according to the Article 167 of Law No. 95/2006, Art. 28, Chapter VIII of Order 904/2006; the EU GCP Directives 2005/28/EC; and the International Conference on Harmonisation of Technical Requirements for Registration of Pharmaceuticals for Human Use.

### 2.2. Inclusion and Exclusion Criteria

Inclusion criteria were the following: (1) adult patients (aged 18 and above); (2) patients with a documented diagnosis of unimalleolar, bimalleolar, or trimalleolar ankle fractures; (3) all fractures were classified according to the International Classification of Diseases (ICD-10) [23] and further categorized using the Danis—Weber and Lauge—Hansen grading systems into specific fracture types (SER, PER, SA, PA); (4) patients were divided into two groups: those who underwent syndesmotic screw fixation as part of their initial fracture treatment (and subsequently had the screw removed) and those treated conservatively without screw fixation (Non-Screw Removal Group); (5) the patients had to be present for evaluation at two time points that were set at 2 and 6 months post-operatively to assess short-term and medium-term outcomes; (6) mandatory post-operative rehabilitation must be undertaken by all participants; (7) decisions to remove or retain the talofibular syndesmotic screw were taken by the patients after careful consideration of the medical advice provided by the orthopedic surgeon.

Exclusion criteria were set for the following issues: (1) patients having incomplete medical records; (2) patients refusing to provide informed consent; (3) patients who developed orthopedic complications, which might confound the study outcomes in terms of quality of life and physical health perception; (4) patients who were lost at follow-up at 2- and 6-months post-screw fixation surgery; (5) patients who did not undergo screw fixation were not included in the current study.

### 2.3. Data Acquisition and Surveys

Extensive demographic and clinical variables were collected. These included age, gender, body mass index, area of residence, marital status, economic standing, educational accomplishments, employment status, and lifestyle habits such as alcohol consumption and smoking habits. Moreover, the Charlson Comorbidity Index (CCI) was employed [24].

Scar tissue from a previous ankle injury can make a ligament stiffer and decrease the range of motion; therefore, both ultrasound assessments and radiographic assessments were carried out to determine the degree of healing after ankle injury involving talofibular diastasis. All participants underwent a rigorous imaging assessment, including elastograms (shear wave elastography) to evaluate ankle dorsiflexion, the anterior talofibular ligament, and the calcaneofibular ligament, as well as evaluations for range of motion (ROM) [25]. Specific measurements related to tibiofibular diastasis, such as TCS—tibiofibular clear space; TOL—tibiofibular overlap; IFD—incisura fibularis depth, were meticulously documented. Results from the ultrasound and radiographic measurements were presented as means with standard deviation for clarity and statistical relevance. 

To assess patient outcomes post-surgery, multiple standardized questionnaires were distributed. These were provided at 2 months and 6 months postoperatively after tibiofibular fixation. The SF-36 Health Survey, renowned for appraising a vast range of health facets from physical to emotional domains, was one of the key tools in the current study [26]. The HADS evaluated data regarding mental health status, discerning levels of anxiety and depression [27]. Furthermore, the WHOQOL-BREF, with its broad spectrum of 26 questions, assessed overall quality of life [28].

### 2.4. Statistical Analysis

Data management and analysis were conducted utilizing the statistical software SPSS version 26.0 (SPSS Inc., Chicago, IL, USA). The sample size was calculated based on a convenience sampling method, with a minimum of 120 respondents at a 95% confidence level and 5% margin of error. Continuous variables were represented as mean ± standard deviation (SD), while categorical variables were expressed in terms of frequencies and percentages. To analyze the differences between two means of continuous variables, the Student’s *t*-test was utilized. The Chi-square test was utilized for the categorical variables. A Pearson and Spearman correlation analysis was performed to determine the degree of association between self-reported patient questionnaires and the objective measurements performed by the physicians. A *p*-value threshold of less than 0.05 was set for statistical significance. To address the potential for Type I errors due to multiple comparisons, Bonferroni correction was applied. The hypothesis-testing approach was guided by the initial research questions, with a focus on exploring specific, pre-defined relationships rather than conducting exploratory analyses. All results were double-checked to ensure accuracy and reliability.

## 3. Results

### 3.1. Patient Demographics

A total of 129 patients were included in the current study after matching the inclusion and exclusion criteria. 26 patients were excluded due to incomplete medical records or being lost at follow-up. 85 patients underwent screw removal, while 44 patients were subjected to the conservative approach without removing the syndesmotic screw. The average age of individuals in the screw removal group was 34.7 years, while that of the conservative approach group was 35.4 years.

When examining the type of fracture, the distribution between unimalleolar, bimalleolar, and trimalleolar fractures was not significantly different between the two groups (*p* = 0.753). Specifically, in the screw removal group, 25.9% had unimalleolar fractures, 52.9% had bimalleolar fractures, and 21.2% had trimalleolar fractures. In the conservative approach group, the distribution was 25.0% for unimalleolar fractures, 45.5% for bimalleolar, and 29.5% for trimalleolar fractures. Lastly, when categorized based on the Lauge–Hansen classification, there were no significant differences between the two groups (*p* = 0.182). In the screw removal cohort, 44.7% had supination external rotation (SER) fractures, 31.8% had pronation external rotation (PER) fractures, 10.6% had supination adduction (SA), and 12.9% had pronation abduction (PA). In the conservative approach group, the distribution was 34.1% for SER, 27.3% for PER, 22.7% for SA, and 15.9% for PA, as described in Table 1.

### 3.2. Ultrasound and Radiographic Measurements

The ankle dorsiflexion shear wave (SW) velocity was significantly lower in the screw removal group (8.9 ± 1.4) compared to the conservative approach group (10.1 ± 1.8, *p* < 0.001), indicating enhanced mobility. Similarly, the anterior talofibular ligament (ATFL) and calcaneofibular ligament (CFL) showed lower SW velocities in the screw removal group (ATFL: 2.8 ± 0.9, CFL: 3.0 ± 1.2) than in the conservative group (ATFL: 3.3 ± 1.3, *p* = 0.012; CFL: 3.6 ± 1.3, *p* = 0.010). Radiographically, the tibiofibular overlap (TOL) and tibiofibular clear space (TCS) also exhibited significant differences, with the screw removal group showing lower values (TOL: 8.0 ± 2.1, TCS: 3.4 ± 0.9) compared to the conservative group (TOL: 8.9 ± 1.6, *p* = 0.014; TCS: 3.9 ± 1.1, *p* = 0.007), suggesting differences in joint fixation quality.

At 6 months post-intervention, the only significant finding was in ankle dorsiflexion SW velocity, which remained lower in the screw removal group (8.6 ± 1.1) compared to the conservative group (9.5 ± 1.3, *p* < 0.001), and in TCS, with a statistically significant difference observed between the screw removal (3.5 ± 1.0) and conservative groups (4.0 ± 1.1, *p* = 0.010). The other measurements, including ATFL and CFL velocities, as well as TOL and incisura fibularis depth (IFD), did not show statistically significant differences at the 6-month mark, as seen in Table 2.

### 3.3. Survey Analysis

**Figure 1:**
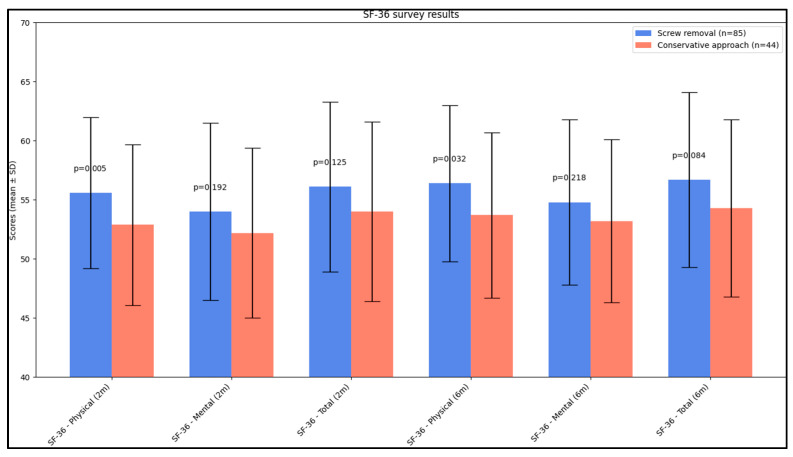
Analysis of SF-36 questionnaire results.

In the survey analysis assessing quality of life, significant findings were noted in the physical health domain. At 2 months post-intervention, the SF-36 physical component score showed a statistically significant difference between the groups. The screw removal group reported a higher physical health score (55.6 ± 6.4) compared to the conservative approach group (52.1 ± 6.8, *p* = 0.005). This suggests a better physical health status among patients who underwent screw removal. At the 6-month follow-up, this trend continued with the screw removal group again reporting a significantly higher physical health score (56.4 ± 6.6) than the conservative approach group (53.7 ± 7.0, *p* = 0.032), although not statistically significant after Bonferroni correction. However, in the mental health component and the total SF-36 score, the differences between the two groups were not statistically significant at either the 2-month or the 6-month mark, respectively. These results imply that while physical health improvements were more pronounced in the screw removal group, mental health outcomes were similar between the two groups over time, as presented in Table 3 and Figure 1.

**Figure 2:**
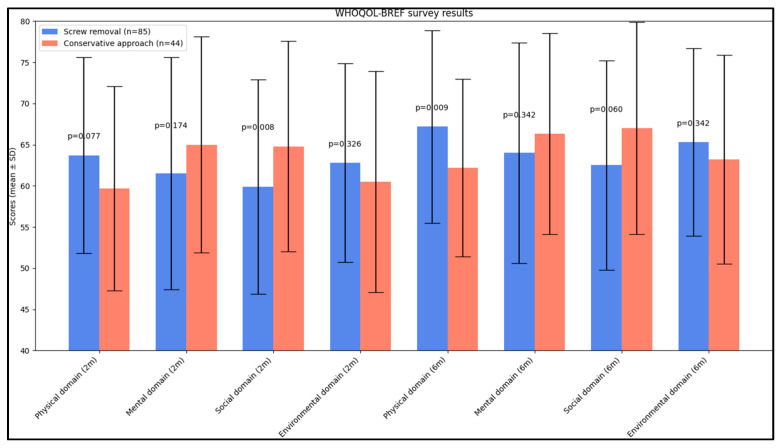
Average values of domain scores on the WHOQOL-BREF questionnaire.

In the WHOQOL-BREF survey results at the 2-month post-intervention mark, a significant difference was observed in the social domain. The conservative approach group scored higher (62.8 ± 12.8) than the screw removal group (59.3 ± 13.0), with a *p*-value of 0.008, indicating a better social quality of life in the conservative approach group at this stage. At the 6-month evaluation, the physical domain showed a significant difference. The screw removal group reported a higher score (67.2 ± 11.7) compared to the conservative approach group (61.6 ± 10.8, *p* = 0.009), suggesting an improvement in physical quality of life among patients who underwent screw removal. The other domains, including the mental and environmental domains, did not show statistically significant differences at either the 2-month or 6-month assessments, indicating similar outcomes in these areas between the two groups over time., as presented in Table 4 and Figure 2.

**Figure 3:**
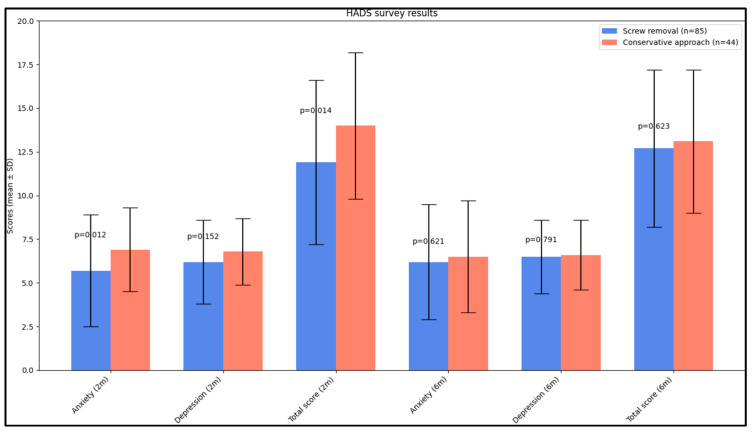
Analysis of HADS questionnaire results.

At the 2-month post-intervention mark, a significant difference was observed in the Hospital Anxiety and Depression Scale (HADS) survey results between the two groups. Patients in the screw removal group reported significantly lower anxiety scores (5.6 ± 2.9) compared to those in the conservative approach group (6.9 ± 2.4, *p* = 0.012). Additionally, the total HADS score, which combines both anxiety and depression scores, was significantly lower in the screw removal group (11.9 ± 4.7) than in the conservative approach group (14.0 ± 4.2, *p* = 0.014), suggesting a better overall mental health status in the screw removal group at this time point. However, by the 6-month post-intervention assessment, these differences in anxiety, depression, and total HADS scores were no longer statistically significant, indicating a convergence in mental health outcomes between the two groups over time, as seen in Table 5 and Figure 3.

In our study, significant correlations were observed between ultrasound and radiographic measurements and self-reported questionnaire scores at both the 2-month and 6-month post-intervention assessments. At the 2-month evaluation, ultrasound measurements of ankle dorsiflexion demonstrated a strong negative correlation with the SF-36 Physical score (r = −0.417). A similar trend was observed between ankle dorsiflexion and the WHOQOL Physical domain (r = −0.394). Interestingly, ankle dorsiflexion was positively correlated with HADS Anxiety (r = 0.312). For the ATFL and CFL velocities, negative correlations with the SF-36 Physical score were also significant (ATFL: r = −0.251; CFL: r = −0.237). In terms of radiographic assessments, TOL and TCS positively correlated with the WHOQOL Physical domain (TOL: r = 0.291; TCS: r = 0.276), and TCS showed a negative correlation with HADS Anxiety (r = −0.228).

**Figure 4:**
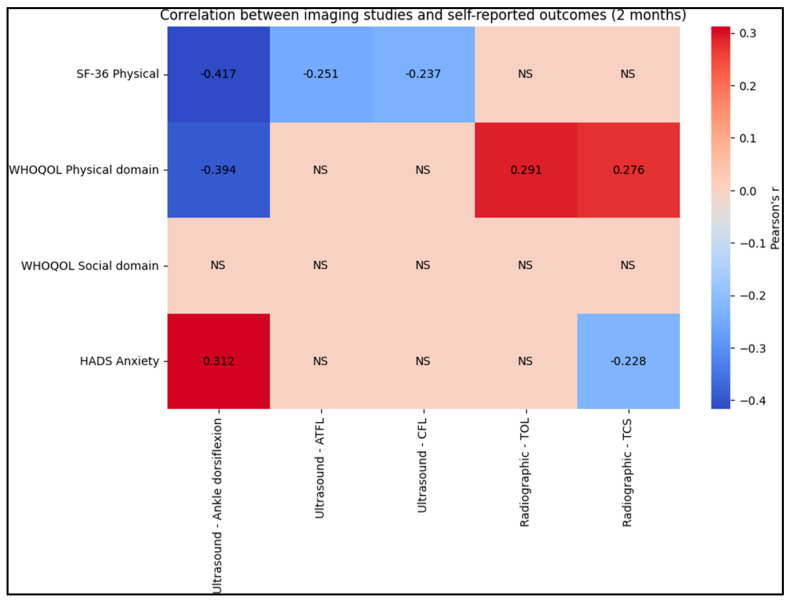
Correlation analysis at 2 months; NS–Not Significant.

**Figure 5:**
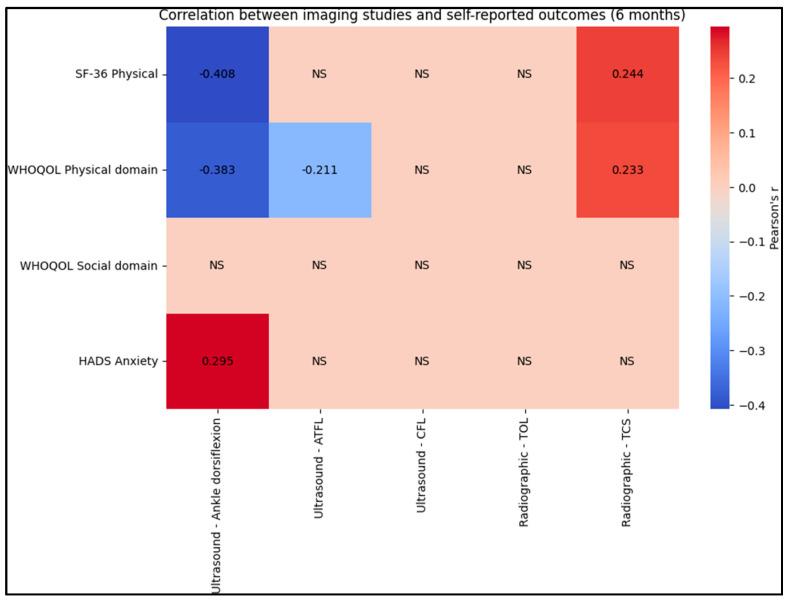
Correlation analysis at 6 months; NS–Not Significant.

By the 6-month mark, the negative correlation between ultrasound ankle dorsiflexion and the SF-36 Physical score remained notable (r = −0.408), as well as with the WHOQOL Physical domain (r = −0.383). The correlation of ankle dorsiflexion with HADS Anxiety was also significant (r = 0.295). Additionally, radiographic TCS continued to show a significant correlation with both the SF-36 Physical (r = 0.244) and the WHOQOL Physical domain (r = 0.233), as presented in Table 6, Figure 4 and Figure 5.

## 4. Discussion

Our study demonstrated significant findings in the realm of ultrasound and radiographic measurements, quality of life assessments, and correlation analysis in patients undergoing screw removal versus a conservative approach for tibiofibular diastasis. Key observations included lower ankle dorsiflexion SW velocities in the screw removal group, indicative of enhanced mobility, and significant improvements in physical health, as reported on the SF-36 survey at both the 2- and 6-month marks. Additionally, the screw removal group reported significantly lower anxiety scores on the HADS survey at the 2-month post-intervention mark. These findings were corroborated by significant correlations between ultrasound measurements of ankle dorsiflexion and both the SF-36 Physical score and the WHOQOL Physical domain, as well as a notable correlation with HADS Anxiety.

The generalizability of our results is supported by existing literature that highlights the importance of functional recovery in orthopedic interventions. While it is widely acknowledged that increased SW velocities indicate reduced mobility [29,30], the differences in the anterior talofibular ligament (ATFL) and calcaneofibular ligament (CFL) velocities further emphasize the biomechanical implications of the chosen intervention [31]. Another recent study determined the ATFL and CFL velocities under SW elastography, obtaining average velocity values under stress of 3.21 for ATFL and 3.42 for CFL, respectively [25], with significantly higher values compared with the at-rest velocities. Therefore, it can be concluded that the choice of intervention may directly influence the viscoelastic properties of these ligaments, which are crucial for ankle stability.

Due to the limitations of clinical examination and traditional radiographs, injuries to the syndesmotic ligaments were frequently overlooked. This misdiagnosis often resulted in inappropriate treatment, leading to lingering issues like instability, pain, and swelling for patients [32,33]. Radiographic metrics, especially tibiofibular overlap (TOL) and tibiofibular clear space (TCS), are essential indicators of the talofibular joint’s fixation quality. Although the differences in TOL and TCS between the two groups were statistically significant at the 2-month mark, these metrics converged by the 6-month assessment. It raises a pertinent question—does the initial post-intervention period hold greater significance in determining long-term outcomes, or do these differences tend to diminish with time and rehabilitation?

Even though in the current research we did not perform an MRI evaluation of the ankle, in the study conducted by JJ. Hermans and colleagues [34], the relationship between radiological assessment of acute ankle fractures and syndesmotic injury as visualized on MRI was explored. The study found that the Lauge—Hansen classification, with a sensitivity and specificity of 92%, was superior in predicting syndesmotic injuries compared to the Weber and AO—Müller systems, which had a sensitivity of 47% and specificity of 100%. The research concluded that MRI was a more precise tool for identifying the severity of syndesmotic injuries and determining the fracture stage than traditional radiographs.

Moreover, it should also be noted that the screw removal group consistently reported slightly superior physical health scores, even if the margin was small. This suggests that the screw removal procedure might offer slight advantages in the domain of physical health recovery, even though there is still an ongoing debate over removing or keeping the syndesmotic screws in place, with different facets of patient outcomes [35,36]. Nevertheless, in the current study, patient outcomes on the mental component scores remained close between the groups, reinforcing the multifactorial nature of mental health recovery post-intervention [37,38]. It is important to consider that the mere perception of undergoing a procedure like screw removal could influence self-reported outcomes due to factors such as patient expectations or perceptions of invasiveness.

The differences in the social domain scores between the groups are intriguing. At 2 months post-intervention, the conservative approach group outperformed the screw removal group, highlighting that the nature of the intervention might influence the patients’ social reintegration. As social interaction and participation can play a pivotal role in overall recovery, this is a domain that warrants further exploration in future research. Moreover, it is worth noting the significantly lower anxiety scores in the screw removal group at the 2-month mark. The etiology of this reduced anxiety is multifaceted, as described in other studies that assessed anxiety levels regarding surgical interventions [39]. The tangible nature of screw removal might provide patients with a sense of finality or resolution, possibly mitigating anxiety levels. However, as both groups trended towards convergence in anxiety and depression scores by the 6-month mark, it suggests that the immediate post-operative phase is critical in influencing psychological outcomes.

In our study, the evaluation of correlations between ultrasound/radiographic measurements and self-reported questionnaire scores constitutes a pivotal aspect, providing critical insights into the interplay between objective clinical findings and subjective patient experiences. The significant correlations observed, particularly the strong negative correlations between ankle dorsiflexion measurements and the SF-36 Physical score, underscore the direct impact of physical mobility on patients’ perceived physical health. Similarly, the positive correlation between ankle dorsiflexion and HADS Anxiety at the 2-month mark highlights the intricate relationship between physical recovery and psychological well-being. These findings are in line with current literature which emphasizes the holistic nature of patient recovery, suggesting that physical improvements post-surgery can have far-reaching effects on mental health and overall quality of life [40,41]. The correlation analysis in our study not only confirms these established notions but also provides a nuanced understanding of how specific clinical interventions like screw removal in tibiofibular diastasis can influence various dimensions of patient health.

In line with our study’s focus on evaluating the outcomes of tibiofibular diastasis treatment, the research conducted by D’Ambrosi et al. [42] on post-traumatic ankle osteoarthritis (PTOA) offers valuable parallels, particularly in the context of quality-of-life assessments. Their study emphasizes the significant impact of PTOA on patients’ daily lives and the correlation between physical and mental health scores and clinical measures like the AOFAS ankle-hindfoot score and VAS pain ratings. This correlation underscores the multifaceted nature of ankle recovery, where physical healing and subjective well-being are closely intertwined, aligning with our findings where different interventions (screw removal vs. conservative) showed varied impacts on both physical and mental health outcomes. Additionally, the work by Efrima B et al. [43], highlighting the reliability of weightbearing CT (WBCT) in ankle diagnostics, complements our study’s emphasis on the utility of imaging in assessing ankle treatment efficacy. The precision of WBCT in evaluating ankle arthroplasty positioning, as demonstrated in their study, suggests a potential future direction for enhancing the accuracy of imaging techniques in tibiofibular diastasis management. These studies collectively reinforce the importance of integrating both objective clinical measures and patient-reported outcomes in the comprehensive assessment of ankle injuries, offering a broader perspective for optimizing treatment strategies in tibiofibular diastasis.

From a medical perspective, the study’s findings underscore the importance of a holistic approach when managing tibiofibular diastasis. While the choice of intervention (screw removal vs. conservative) has evident implications on biomechanics and radiographic outcomes, the psychological and social aspects of recovery are equally consequential. The variability in outcomes across domains highlights the need for tailored post-operative care plans, addressing not just the physical, but also the psychological and social facets of rehabilitation.

The study’s demonstration of a significant correlation between imaging findings and patient-reported outcomes provides clinicians with a more comprehensive approach to evaluating the effectiveness of tibiofibular diastasis treatments, aiding in the tailoring of patient-specific management strategies. Particularly, the insights into the differences in short-term and long-term outcomes between surgical and conservative approaches offer guidance for decision-making in treatment planning, emphasizing the importance of considering both physical and mental health parameters in patient care.

The current research offers significant insights into the outcomes of patients with varying types of ankle fractures. However, there are several limitations to consider, such as the study being confined to a single medical center in Romania, which might limit the generalizability of our findings to other populations or healthcare settings. The rigorous criteria for inclusion and exclusion, while ensuring data quality, may omit certain patient groups or complications that could be pertinent to a more comprehensive understanding of the condition. It should also be noted that while the study meticulously used the Danis—Weber and Lauge—Hansen grading systems, there are potential human errors in classification. Moreover, despite the use of sophisticated imaging assessments, like elastograms, radiographic interpretations inherently have subjective elements that may introduce bias. Lastly, while standardized questionnaires like the SF-36, HADS, and WHOQOL-BREF provide a comprehensive view of the patient’s well-being, the inherent self-reporting nature of these tools can be influenced by recall bias, personal perceptions, or cultural factors, potentially influencing the accuracy of reported post-operative outcomes.

## 5. Conclusions

The findings of this study demonstrate a significant relationship between imaging outcomes and self-reported quality-of-life measures in patients with tibiofibular diastasis, aligning with the study’s purpose of exploring these associations. We found that screw removal leads to improved physical outcomes as evidenced by lower shear wave velocities and better radiographic metrics, indicative of enhanced mobility and joint stability. These improvements are reflected in the short-term reduction of anxiety levels. However, the study also reveals that mental health parameters, specifically anxiety and depression, converge between treatment groups by the 6-month mark. These insights provide valuable evidence to clinicians for making informed decisions in managing tibiofibular diastasis, emphasizing the need to consider both physical and psychological aspects of patient recovery.

## Figures and Tables

**Table 1:** Comparison of the study cohort background characteristics.

Variables	Screw Removal (*n* = 85)	Non Screw Removal (*n* = 44)	*p*-Value *
Age, years	34.7 ± 13.3	35.4 ± 14.8	0.786
Sex (men, %)	51 (60.0%)	20 (45.5%)	0.092
Overweight (>25.0 kg/m^2^)	33 (38.8%)	21 (47.7%)	0.327
Smoking	22 (25.9%)	15 (34.1%)	0.392
CCI > 2	7 (8.2%)	3 (6.8%)	0.542
Fracture type			0.753
Unimalleolar	22 (25.9%	11 (25.0%)	
Bimalleolar	45 (52.9%)	20 (45.5%)	
Trimalleolar	18 (21.2%)	13 (29.5%)	
Lauge–Hansen classification			0.182
SER	38 (44.7%)	15 (34.1%)	
PER	27 (31.8%)	12 (27.3%)	
SA	9 (10.6%)	10 (22.7%)	
PA	11 (12.9%)	7 (15.9%)	

* Chi-square or Fisher’s exact test, with a significance threshold of 0.006 after Bonferroni correction; CCI—Charlson Comorbidity Index; SER—supination external rotation fracture; PER—pronation external rotation fracture; SA—Supination adduction; PA—Pronation abduction.

**Table 2:** Imaging studies at 2 months and 6 months post-intervention between patients with removed talofibular syndesmotic screw and those with conservative management.

Variables	Screw Removal (*n* = 85)	Non Screw Removal (*n* = 44)	*p*-Value *
At 2 months			
Ultrasound (SW velocity **)			
Ankle dorsiflexion	8.9 ± 1.4	10.1 ± 1.8	<0.001
ATFL	2.8 ± 0.9	3.3 ± 1.3	0.012
CFL	3.0 ± 1.2	3.6 ± 1.3	0.010
Radiographic changes ***			
TOL	8.0 ± 2.1	8.9 ± 1.6	0.014
TCS	3.4 ± 0.9	3.9 ± 1.1	0.007
IFD	4.0 ± 1.2	4.3 ± 0.8	0.138
At 6 months			
Ultrasound (SW velocity)			
Ankle dorsiflexion	8.6 ± 1.1	9.5 ± 1.3	<0.001
ATFL	2.6 ± 0.9	2.9 ± 1.0	0.086
CFL	2.8 ± 1.3	3.1 ± 0.9	0.173
Radiographic changes			
TOL	8.3 ± 1.6	8.8 ± 2.0	0.125
TCS	3.5 ± 1.0	4.0 ± 1.1	0.010
IFD	4.1 ± 1.5	4.4 ± 1.2	0.252

* Student’s *t*-test, with a significance threshold of 0.016 after Bonferroni correction; **—Higher values are associated with decreased mobility and ROM); ***—Higher values are associated with better fixation of the talofibular joint; ROM—range of motion; TCS—tibiofibular clear space; TOL—tibiofibular overlap; IFD—incisura fibularis depth; SD—standard deviation; SW—shear wave; ATFL—anterior talofibular ligament; CFL—calcaneofibular ligament.

**Table 3:** SF-36 survey results.

Scores (Mean ± SD)	Screw Removal (*n* = 85)	Non Screw Removal (*n* = 44)	*p*-Value *
At 2 months			
SF-36—Physical	55.6 ± 6.4	52.1 ± 6.8	0.005
SF-36—Mental	54.0 ± 7.5	52.2 ± 7.2	0.192
SF-36—Total	56.1 ± 7.2	54.0 ± 7.6	0.125
At 6 months			
SF-36—Physical	56.4 ± 6.6	53.7 ± 7.0	0.032
SF-36—Mental	54.8 ± 7.0	53.2 ± 6.9	0.218
SF-36—Total	56.7 ± 7.4	54.3 ± 7.5	0.084

* Student’s *t*-test, with a significance threshold of 0.016 after Bonferroni correction; SD—standard deviation; SF-36—short form survey (higher scores indicate better health status and quality of life).

**Table 4:** WHOQOL-BREF survey results.

WHOQOL-BREF (Mean ± SD)	Screw Removal (*n* = 85)	Non Screw Removal (*n* = 44)	*p*-Value *
At 2 months			
Physical domain	63.7 ± 11.9	59.7 ± 12.4	0.077
Mental domain	61.5 ± 14.1	65.0 ± 13.1	0.174
Social domain	59.3 ± 13.0	65.8 ± 12.8	0.008
Environmental domain	62.8 ± 12.1	60.5 ± 13.4	0.326
At 6 months			
Physical domain	67.2 ± 11.7	61.6 ± 10.8	0.009
Mental domain	64.0 ± 13.4	66.3 ± 12.2	0.342
Social domain	62.5 ± 12.7	67.0 ± 12.9	0.060
Environmental domain	65.3 ± 11.4	63.2 ± 12.7	0.342

* Student’s *t*-test, with a significance threshold of 0.012 after Bonferroni correction; SD—standard deviation; WHOQOL-BREF—brief version of the World Health Organization Quality of Life survey (higher scores indicate better quality of life).

**Table 5:** HADS survey results.

HADS (Mean ± SD)	Screw Removal (*n* = 85)	Non Screw Removal (*n* = 44)	*p*-Value *
At 2 months			
Anxiety	5.6 ± 2.9	6.9 ± 2.4	0.012
Depression	6.2 ± 2.4	6.8 ± 1.9	0.152
Total score	11.9 ± 4.7	14.0 ± 4.2	0.014
At 6 months			
Anxiety	6.2 ± 3.3	6.5 ± 3.2	0.621
Depression	6.5 ± 2.1	6.6 ± 2.0	0.791
Total score	12.7 ± 4.5	13.1 ± 4.1	0.623

* Student’s *t*-test, with a significance threshold of 0.016 after Bonferroni correction; SD—standard deviation; SF-36—short form survey (higher scores indicate higher levels of anxiety or depression).

**Table 6:** Correlation analysis between self-reported questionnaires and objective measurements.

Variables	Pearson’s r	*p*-Value
At 2 months		
Ultrasound—Ankle dorsiflexion vs. SF-36 Physical	−0.417	<0.001
Ultrasound—Ankle dorsiflexion vs. WHOQOL Physical domain	−0.394	0.003
Ultrasound—Ankle dorsiflexion vs. HADS Anxiety	0.312	0.027
Ultrasound—ATFL vs. SF-36 Physical	−0.251	0.019
Ultrasound—CFL vs. SF-36 Physical	−0.237	0.023
Radiographic—TOL vs. WHOQOL Physical domain	0.291	0.016
Radiographic—TCS vs. WHOQOL Physical domain	0.276	0.021
Radiographic—TCS vs. HADS Anxiety	−0.228	0.033
At 6 months		
Ultrasound—Ankle dorsiflexion vs. SF-36 Physical	−0.408	<0.001
Ultrasound—Ankle dorsiflexion vs. WHOQOL Physical domain	−0.383	0.004
Ultrasound—Ankle dorsiflexion vs. HADS Anxiety	0.295	0.038
Ultrasound—ATFL vs. WHOQOL Physical domain	−0.211	0.054
Radiographic—TCS vs. SF-36 Physical	0.244	0.026
Radiographic—TCS vs. WHOQOL Physical domain	0.233	0.031

SF—short form; WHOQOL—World Health Organization Quality of Life; HADS—Hospital Anxiety and Depression score; TCS—tibiofibular clear space; TOL—tibiofibular overlap; ATFL—anterior talofibular ligament; CFL—calcaneofibular ligament.

## Data Availability

Data available on request.
